# Improving the Precision of Ability Estimates Using Time-On-Task Variables: Insights From the PISA 2012 Computer-Based Assessment of Mathematics

**DOI:** 10.3389/fpsyg.2021.579128

**Published:** 2021-03-19

**Authors:** Denise Reis Costa, Maria Bolsinova, Jesper Tijmstra, Björn Andersson

**Affiliations:** ^1^Centre for Educational Measurement, Faculty of Educational Sciences, University of Oslo, Oslo, Norway; ^2^Methodology and Statistics, Tilburg University, Tilburg, Netherlands

**Keywords:** log files, computer-based assessment, time on task, measurement precision, measurement invariance, PISA

## Abstract

Log-file data from computer-based assessments can provide useful collateral information for estimating student abilities. In turn, this can improve traditional approaches that only consider response accuracy. Based on the amounts of time students spent on 10 mathematics items from the PISA 2012, this study evaluated the overall changes in and measurement precision of ability estimates and explored country-level heterogeneity when combining item responses and time-on-task measurements using a joint framework. Our findings suggest a notable increase in precision with the incorporation of response times and indicate differences between countries in how respondents approached items as well as in their response processes. Results also showed that additional information could be captured through differences in the modeling structure when response times were included. However, such information may not reflect the testing objective.

## 1. Introduction

Computers have become increasingly common implements used in classroom activities over the past few decades. As a reflection of this trend, large-scale educational assessments have moved from paper and pencil based tests to administrated computer assessments. In addition to being more efficient and reducing human error, computer-based assessments allow for a greater variety of tasks. Further, interactive computer environments can be used to generate log files, which provide easy access to information concerning the examinee response process. These log files contain time-stamped data that provide a complete overview of all communication between the user-interface and server (OECD, 2019). As such, it is possible to trace how respondents interact with the testing platform while gathering information about the amount of time spent on each task.

The first computer-based administration of the Programme for International Student Assessment (PISA) dates back to 2006 (OECD, 2010). However, more extensive studies involving log files were enabled through the release of the PISA 2009 digital reading assessment (OECD, 2011). In this context, time-on-task and navigating behaviors can be extracted from these log files as relevant variables. The information derived from variables of this type can help teachers further understand the solution strategies used by students while also enabling a substantive interpretation of respondent-item interactions (Greiff et al., 2015; Goldhammer and Zehner, 2017). The variables taken from log files can also be included in sophisticated models designed to improve student proficiency estimations (van der Linden, 2007).

While log file data from computer-based assessments have been available for several years, few studies have investigated how they can be used to improve the measurement precision of resulting scores. Using released items from the 2012 PISA computer-based assessment of mathematics, this study thus explored the potential benefits of incorporating time-on-task variables when estimating student proficiency. We specifically compared three different models to advance the current understanding of what time-on-task adds to scores resulting from an international large-scale assessment program.

### 1.1. Time-On-Task and Item Responses

Several previous studies have investigated the relationship between time-on-task and item responses. For example, Goldhammer et al. (2015) studied the relationship between item responses and response times through a logical reasoning test, thus finding a non-linear relationship between reasoning skills and response times. Further, Goldhammer and Klein Entink (2011) investigated how time-on-task and item interactivity behaviors were related to item responses using complex problem-solving items. In addition, Naumann and Goldhammer (2017) found a non-linear relationship between time on task and performance on digital reading items from the PISA 2009 assessment. Finally, Goldhammer et al. (2014) studied the relationship between time-on-task, reading, and problem solving using PIAAC data. Results indicated that the association between time-on-task and performance varied from negative to positive depending on the subject matter and type of task.

In large-scale educational assessments, student proficiency is mainly estimated through the item response theory (IRT) framework (von Davier and Sinharay, 2013). Here, categorical item-response data are considered manifestations of an underlying latent variable that is interpreted as, for example, mathematics proficiency. While time-on-task can be incorporated in several different ways from an IRT perspective (van der Linden, 2007), the state-of-the-art view considers them as realizations of random variables, much like actual item responses (Kyllonen and Zu, 2016). A hierarchical model is most commonly used with time-on-task data. Specifically, a two-level structure is used to incorporate time-on-task, item responses, and latent variables into a single model (van der Linden, 2007). While the hierarchical modeling framework has the advantage of considering both response accuracy and response times as latent variables, it has practical limitations in that it requires specialized software for fitting the model. Molenaar et al. (2015) illustrated how the hierarchical model can be slightly simplified such that standard estimation techniques could be used. This type of formulation of the model allows the use of both generalized linear latent variable models (Skrondal and Rabe-Hesketh, 2004) and non-linear mixed models (Rijmen et al., 2003) with item-response and time-on-task data. Furthermore, the approach outlined by Molenaar et al. (2015) encompasses not only the standard hierarchical model (with the necessary simplification) but also its extensions which allow for more complex relationship between time-on-task and ability such as the model of Bolsinova and Tijmstra (2018). For these reasons, this study pursued the approach of Molenaar et al. (2015) for its analysis of PISA 2012 data.

### 1.2. The Present Study

This study investigated the utility of combining item responses with time-on-task data in the context of a large-scale computer-based assessment of mathematics. It also evaluated the properties of the employed model with respect to each participating country^1^. Specifically, the framework developed by Molenaar et al. (2015) was used to investigate how measurement precision was influenced by incorporating item responses and time-on-task data into a joint model. We also explored country-level heterogeneity in the time-on-task measurement model. As such, the model proposed for this analysis of computer-based large-scale educational assessments implied a different set of underlying assumptions than current procedures. Specifically, we viewed response-time data as comprising an extra information set that enabled us to gain additional insight regarding the latent construct of interest. This also implies that any inference regarding the underlying construct at the country level would potentially change through the proposed approach as opposed to current analysis methods, which this study also investigated. The three following research questions were thus proposed:

- RQ1: What changes occur in the overall ability estimates and their level of precision regarding PISA 2012 digital mathematics items when time-on-task data are included in the analysis?
- RQ2: How do time-on-task model parameters differ across items and countries?
- RQ3: What changes occur in country-level performance when time-on-task data are considered in the analysis?

Our findings should add to the current literature on the relationship between time-on-task and responses to performance items. Our results also have important implications for large-scale assessment programs in regard to evaluating the added measurement precision that is granted by incorporating additional data sources (e.g., time-on-task). Such investigations can inform large-scale assessment programs about whether and how time-on-task data should be included in models designed to generate operational results reports.

## 2. Data and Methods

### 2.1. The 2012 PISA Computer-Based Assessment of Mathematics

PISA administered its first computer-based mathematics literacy assessment as part of its fifth program edition. A total of 32 countries participated in this effort. In this context, 40 min were allocated for the computer-based portion of the test, with math items arranged in 20 min clusters that were assembled with digital reading or problem-solving prompts (OECD, 2014a). A total of 41 math items were selected for this assessment. These items varied from standard multiple-choice to constructed response formats.

Table 1 presents the characteristics of the PISA sample by country (sample size, Math performance, and variation) for the whole computer-based of mathematics clusters (41 items) as well as to the subsample with available and valid log-file data (10 items).

**Table 1:** Sample size, mean score, and variation in student performance on all clusters, as well as sample size, percentage of female, average total time, and percentage of missing responses for the 10 released and valid log-file data from the PISA 2012 computer-based mathematics by country.

**Country**	**All clusters (41 items)**			**Valid log-file data (10 items)**			
	***n***	**Mean**	**S.D**.	***n***	**% Female**	**Average total time (min)**	**% Missing response**
SGP	2,873	566.02	98.34	453	49.89	16.13	3.22
QCN	2,409	562.26	93.64	393	49.87	16.13	0.64
KOR	2,675	552.57	90.15	433	44.34	13.92	1.20
HKG	2,714	549.64	86.71	421	45.37	15.37	2.45
MAC	3,147	542.90	82.85	522	50.00	17.92	3.41
JPN	6,351	539.01	87.80	982	46.44	15.65	3.21
TAP	3,063	537.26	88.80	513	51.27	15.13	2.51
CAN	10,817	522.85	91.92	1,527	51.34	14.28	4.16
EST	2,837	516.09	82.13	460	50.00	14.54	2.37
BEL	4,617	512.15	98.60	707	49.50	14.19	4.82
DEU	2,881	509.37	95.50	441	51.02	13.75	2.43
FRA	3,012	508.06	91.95	440	53.18	15.43	4.41
AUS	11,834	507.70	90.94	1,833	48.88	13.55	1.99
AUT	2,731	507.34	88.74	436	50.92	13.42	1.28
ITA	3,089	498.76	83.14	440	45.68	16.54	6.86
USA	2,572	498.03	88.75	402	46.77	14.57	1.89
NOR	2,924	497.56	87.25	413	48.67	13.48	2.20
SVK	3,145	497.34	86.07	505	44.75	16.24	5.88
DNK	4,149	496.19	86.41	629	51.83	13.51	1.43
IRL	2,613	493.08	80.50	389	51.41	14.85	3.26
SWE	2,671	489.93	86.06	423	52.48	13.84	3.62
RUS	3,186	489.15	79.83	531	50.28	16.36	4.24
POL	2,567	489.04	86.01	428	52.10	13.09	1.64
PRT	3,272	489.03	85.09	487	48.05	15.52	3.29
SVN	4,385	486.94	87.83	678	45.87	10.95	0.65
ESP	5,751	475.08	81.99	933	50.38	14.44	3.63
HUN	2,746	469.84	92.58	445	52.81	12.79	1.82
ISR	2,677	446.61	111.28	387	54.78	14.65	2.48
ARE	6,732	434.06	84.28	1057	51.09	14.03	4.07
BRA	3,172	420.74	83.85	480	50.00	16.40	9.92
COL	5,173	396.84	73.33	782	53.58	16.48	8.09
Overall mean	3,961			612	49.77	14.62	3.40

*(1) Countries are displayed by the ISO three-letter code. Their correspondence names are available at the Supplementary Material. (2) Countries are sorted by mean scores. (3) Mean scores and variation were retrieved by OECD (2014c) report, Annex B3. OECD does not provide the overall mean quantities. Remaining statistics were calculated by the authors using PISA 2012 micro and log-file data*.

We utilized data from a total of 18,970 students across 31 countries. We excluded data from Chile since log-file data for two of the analyzed items were unavailable (I20Q1 and I20Q3). Students with invalid information (e.g., those that did not receive final scores or had incomplete timing information) were also excluded from the analysis. On average, the sample size of each country is around 600 (S.D.= 333), the percentage of female is 50% across all the countries. The average total time on the 10 items varied from 10.95 to 17.92 min. Brazil was the country with the highest percentage of missing responses (9.92%) on the analyzed items.

The analyzed log-file data from 10 items were made publicly available on the OECD website. We thus extracted the time students spent on analyzed items and their final responses (i.e., response accuracy). All items were allocated in three units (CD production: items “I15Q1,” “I15Q2,” and “I15Q3”; Star points: items “I20Q1,” “I20Q2,” “I20Q3,” and “I20Q4”; Body Mass Index: items “I38Q3,” “I38Q5,” and “I38Q6”) and were administered in the same cluster.

Table 2 shows the reported item characteristics by OECD (international percent of correct responses, and thresholds used for scaling the items in PISA 2012) as well as the average response time and percentage of missing responses by item.

**Table 2:** Characteristics of the released PISA 2012 computer-based of mathematics items.

**Item**	**Format**	**% correct**	**Thresholds - PISA scale**		**Average response time (min)**	**% Missing response**
			**1**	**2**		
I15Q1	MC	59.02	498.51		1.36	0.81
I15Q2	CR	8.43	685.84	700.72	1.87	0.93
I15Q3	CR	29.02	577.18	658.58	1.98	3.25
I20Q1	CR	29.58	562.07	690.91	2.18	1.69
I20Q2	MC	47.42	549.29		0.96	1.93
I20Q3	CR	26.91	644.25		1.33	2.55
I20Q4	MC	44.12	565.73		0.84	3.30
I38Q3	MC	67.13	468.75		1.25	3.94
I38Q5	CR	27.75	641.05		1.82	6.60
I38Q6	CR	23.24	660.45		1.56	8.96

*(1) Item are displayed by the position within the cluster. (2) MC = Multiple Choice and CR= Constructed Response item type. (3) The international percent of correct responses, and thresholds values were retrieved by OECD (2014b) report, Annex A1. Remaining statistics were calculated by the authors using PISA 2012 log-file data. (4) Threshold values in PISA 2012 were defined as the ability at which the probability of achieving that score or higher reaches 0.62 using a partial credit model*.

Although the effects of the item position were likely negligible due to the length of the computer-based assessment (OECD, 2014b), we were still able to determine that the percentages of missing data were larger for items located at the end of the cluster. We used the full information maximum likelihood approach (FIML) featured in Mplus version 7.3 (Muthén and Muthén, 2012) to incorporate all available data into our analyses. Doing this, the missing responses were treated as missing at random (MAR) and all the available data were incorporated in the modeling.

### 2.2. Statistical Analyses

This study compared three measurement models to estimate student proficiency based on the abovementioned PISA dataset. All these models can be seen as special cases of the framework of Molenaar et al. (2015). They are:

- Model 1 (M1): It provided a baseline and thus only included response accuracy in a unidimensional IRT framework. The model can be seen as a special case of the framework of Molenaar et al. (2015) in which it is assumed that there is no relationship between latent proficiency and response time data.
- Model 2 (M2): A multidimensional latent variable model for the response accuracy and response times, where the response accuracy are related to a latent proficiency and the response times are related to a latent speed. The latent factors are assumed to be correlated. This is a variant of the model described in Molenaar et al. (2015): Here the relationship between the latent proficiency and response times is specified through the relationship between the latent proficiency and latent speed.
- Model 3 (M3): A multidimensional latent variable model for the response accuracy and response times, where response accuracy is related to a latent proficiency and the response times are related to a latent speed and proficiency. This is also a special case of the approach of Molenaar et al. (2015) in which the relationship between latent proficiency and response times goes not only through the relationship between latent proficiency and latent speed, but also through the direct relationship between the ability and individual response times. For this model we employed a particular rotation approach described in Bolsinova and Tijmstra (2018).

**Figure 1:**
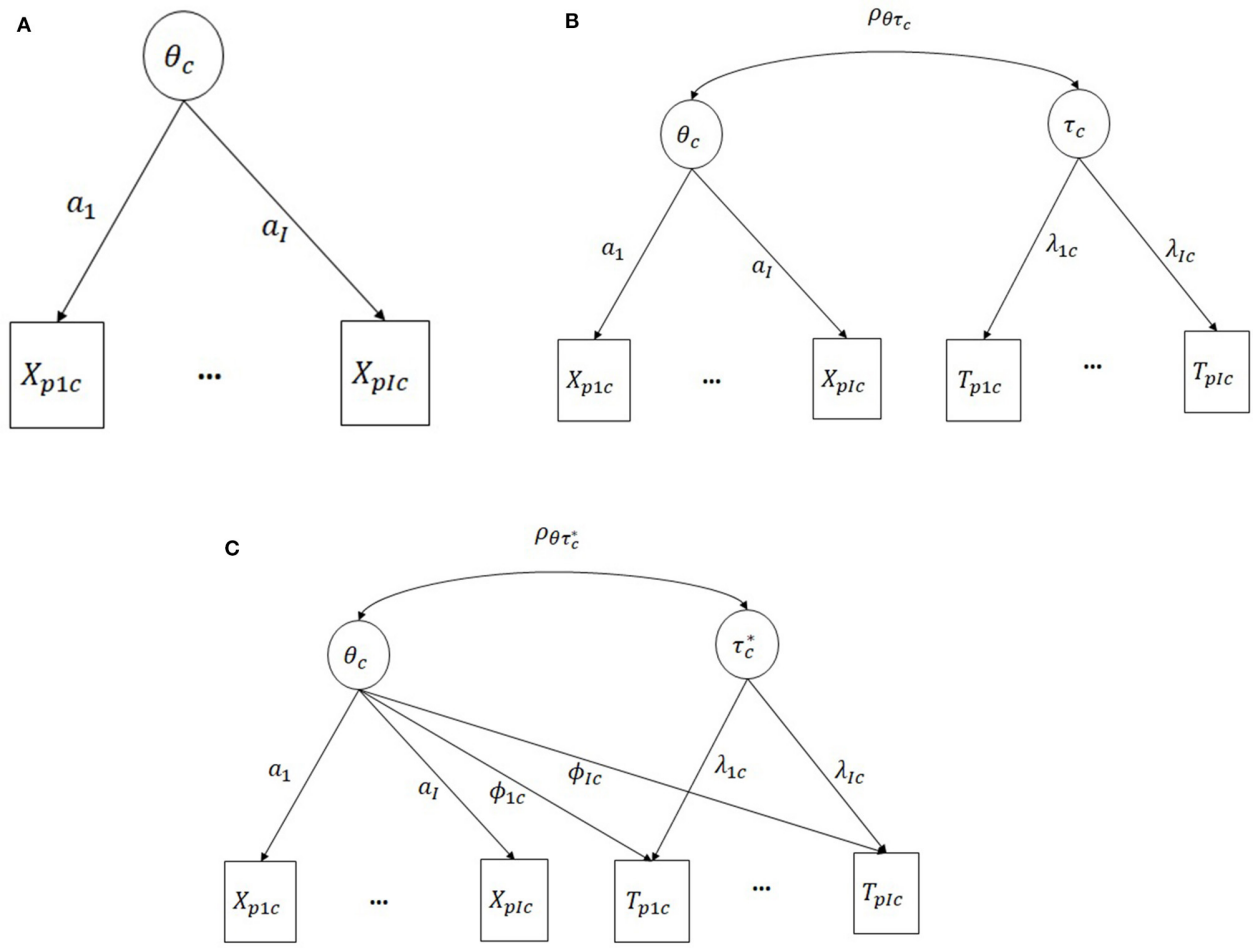
**(A)** M1: response accuracy only **(B)** M2: simple-structure hierarchical model **(C)** M3: Extended hierarchical model with cross-loadings. The parameter's sub-indices are: p, person; I, item; c, country.

Figure 1 shows the graphical representation of the models across PISA countries. For comparability purposes, the items' parameters for response accuracy were fixed from model 1 into models 2 and 3. This approach assures that the models are on the same scale since the relationship between response accuracy and latent proficiency will be the same across models.

This section discusses the mathematical formulations used in each model. The steps used to estimate model parameters for use with the PISA dataset and an analysis of measurement invariance across countries are discussed later.

#### 2.2.1. Model Specification

Let **X** = (*X*_1_, …, *X*_*I*_) be a random vector of responses on the *I* items and **T** = (*T*_*p*1_, …, *T*_*pI*_) be a random vector of response times on the same items with realizations **x**_*p*·_ = (*x*_*p*1_, …, *x*_*pI*_) and **t**_*p*·_ = (*t*_*p*1_, …, *t*_*pI*_), for each person *p*, respectively.

For response accuracy, we adopted the graded response model (GRM) used by Samejima (1969). This was done because some PISA items used a partial scoring method and, unlike other IRT models used for polytomous data (e.g., the partial credit model), the GRM is equivalent to simple factor analytic models in application to discrete data and can therefore be fitted using standard factor analysis software and structural equation models. The differences between the various IRT models used for polytomous data are usually very small; in our case, only three items out of 10 allowed partial scoring. The GRM specifies the conditional probability to obtain each category *k* ∈ [1:*m*], where *m* is the highest possible category for the item. The conditional probability of obtaining this score or higher, given the latent trait θ, is defined by

(1) $$\begin{array}{r} \mathrm{Pr}\left(X_{i}\geq k|\theta \right)=\frac{\exp\left[a_{i}\left(\theta -b_{ik}\right)\right]}{1+\exp\left[a_{i}\left(\theta -b_{ik}\right)\right]}, \end{array}$$

where *a*_*i*_ is the item factor loading/discrimination parameter, and *b*_*ik*_ is the item category threshold parameter^2^. The probability of obtaining a particular response category *k* is then

(2) $$\begin{array}{r} \mathrm{Pr}\left(X_{i}=k|\theta \right)=\mathrm{Pr}\left(X_{i}\geq k|\theta \right)-\mathrm{Pr}\left(X_{i}\geq k+1|\theta \right), \end{array}$$

where Pr(*X*_*i*_ ≥ 0) = 1 and Pr(*X*_*i*_ ≥ *m* + 1) = 0. When *m* = 2, the GRM reduces to the two-parameter logistic IRT model used by Birnbaum (1968), with only one difficulty parameter *b*_*i*_ per item instead of multiple threshold parameters. M1 defined exclusively by Equation (2).

There are also cases in which both responses and response times are used to estimate respondent proficiency. Here, instead of simply specifying the model for response accuracy, we must specify the full model for the joint distribution of response accuracy and response times. For Model 2, we thus adopted the hierarchical modeling approach used by van der Linden (2007), which requires not only the specification of the measurement model for response accuracy (in our case, the GRM) but also the specification of the measurement model for the response times, and the models for the relationship of the latent variables in the two measurement models. The model used for the relationship between item parameters in the two measurement models is often specified, as well. However, as shown by Molenaar et al. (2015), excluding this relationship does not substantially change the parameter estimates, especially when large sample sizes are involved. Furthermore, the use of standard estimation techniques is prevented when including a model for the item parameters. Given the very large sample sizes available in this analysis, we thus specified a higher-order relationship on the person side (i.e., the model for latent variables), but did not do so on the item side.

The joint distribution of response accuracy and response times is conditional to both latent proficiency and speed (denoted by τ) in the hierarchical model. In this case, it is assumed to be a product of the marginal distribution of response accuracy, which only depends on latent proficiency, and the marginal distribution of response time, which only depends on latent speed. We refer to this as a simple-structure model because every observed variable therein is solely related to one latent variable. This differs from the extension of the hierarchical model used by Bolsinova and Tijmstra (2018), which includes direct relationships between response times and latent proficiency in addition to its relation to latent speed.

A lognormal model with item-specific loadings was used for the response times (Klein Entink et al., 2009). It is equivalent to the one-factor model used for log-transformed response times. The conditional distribution of response time on item *i* given the latent speed variable is defined by

(3) $$\begin{array}{r} T_{i}\sim \ln\;N\left(\xi_{i}-\lambda_{i}\tau ,\sigma_{i}^{2}\right), \end{array}$$

which is the lognormal distribution in which the mean is dependent on the item time intensity ξ_*i*_ and the latent speed τ. The strength of the relationship between the response time and the latent speed depends on the factor loading λ_*i*_. Meanwhile, $\sigma_{i}^{2}$ denotes the item-specific residual variance.

The dependence between the latent proficiency and the latent speed variables is modeled using a bivariate normal distribution with correlation parameter ρ. This correlation between the latent variables specifies the indirect relationship between response times and latent proficiency. In turn, this allows us to strengthen the measurement of proficiency (i.e., increase measurement precision) by using the information contained in the response times. The magnitude of the improvement in measurement precision is solely determined by the size of the correlation between the latent speed and latent proficiency (Ranger, 2013).

M3 employed the same model for response accuracy as that used in M1 and M2. However, a different model was used for response times. That is, the mean of the lognormal distribution of response time was dependent on two latent variables, as follows:

(4) $$\begin{array}{r} T_{i}\sim \ln\;N\left(\xi_{i}-\lambda_{i}\tau^{*}+\phi_{i}\theta ,\sigma_{i}^{2}\right), \end{array}$$

where the cross-loading ϕ_*i*_ specifies the strength of the relationship between response time and proficiency. Here, an asterisk is used for the latent variable τ^*^ because it should be interpreted differently from the simple-structure model (M2). Since the cross-loadings between latent proficiency and response time are freely estimated, the correlation between θ and τ^*^ is not identified and is instead fixed to zero so that τ^*^ can be interpreted as a latent variable, thus explaining the covariance of the response times that cannot be explained by latent proficiency. However, it is possible to rotate the latent variable τ^*^ to match the latent speed variable of the simple-structure model. Following Bolsinova and Tijmstra (2018), we will apply a rotation of the factors such that τ^*^ is the latent variable that explains most of the variance of response times. In that case, the correlation between latent proficiency and speed and the corresponding values for the transformed factor loading in the two dimensions can be calculated.

#### 2.2.2. Analysis Strategies

We used the LOGAN R package version 1.0 (Reis Costa and Leoncio, 2019) to extract student response times and accuracy from the PISA 2012 log file containing data for 10 digital math items. We then conducted analyses according to two steps.

First, we fitted all three models by combining the sample consisting of 31 countries to estimate model parameters at an international level. Then, we analyzed the models across PISA countries by fixing specific parameters from previous analyses to allow cross-model comparisons. We also evaluated parameter invariance in the response time model. All model parameters were estimated using the restricted maximum likelihood method in Mplus version 7.3 (Muthén and Muthén, 2012).

Table 3 summarizes the analytical framework used in the first step. Item discrimination (*a*_*i*_s) and threshold parameters (*b*_*i*_s) were freely estimated for Model 1, with the proficiency mean (μ_θ_) and variance ($\sigma_{\theta }^{2}$) fixed to 0 and 1, respectively. To enable model comparisons, the item discrimination and threshold parameters were not estimated for M2 and M3 but were rather fixed to the parameter estimates from M1. For these models, the response time parameters (ξ_*i*_s, λ_*i*_s, $\sigma_{i}^{2}$s, and ϕ_*i*_s) and the mean and variance of the proficiency were freely estimated.

**Table 3:** Framework for the estimation of international parameters for each analyzed model.

**Model**	**μ_θ_**	**$\sigma_{\theta }^{2}$**	**μ_*τc*_**	**$\sigma_{\tau c}^{2}$**	***a*_*i*_s**	***b*_*i*_s**	**ξ_*i*_s**	**λ_*i*_s**	**$\sigma^{2_{i}}$s**	**ρ_*θτ*_**	**ϕ_*i*_s**
M1	0	1	-	-	Free	Free	-	-	-	-	-
M2	Free	Free	0	1	*a*_*i*_s M1	*b*_*i*_s M1	Free	Free	Free	Free	-
M3	Free	Free	0	1	*a*_*i*_s M1	*b*_*i*_s M1	Free	Free	Free	0	Free

For M3, the second latent variable (τ^*^) was rotated to obtain the estimates of the transformed factor loadings (i.e., the factor loadings for speed correspond to the relationship between response time and the latent variable which has the same interpretation as τ in M2).

All analyses were conducted assuming the same graded response model for the item-response modeling. We evaluated the fit of the GRM model for M1 (in which the item discrimination and threshold parameters were freely estimated) by calculating two approximate fit statistics [i.e., the Root Mean Square Error of Approximation (RMSEA) and the Standardized Root Mean Square Residual (SRMR)] using the complete dataset in the mirt R package (Chalmers, 2012). As a guideline, cutoff value close to 0.08 for SRMR and a cutoff value close to 0.06 for RMSE indicated acceptable fit (Hu and Bentler, 1999).

We conducted country-level analyses in the second step. Table 4 shows the fixed and freely estimated parameters for each model. Here, models containing the suffix “_Full” indicate full measurement invariance. That is, we estimated each country's mean and variance for the latent variables (θ_*c*_, τ_*c*_, or ρ_θ*τc*_), fixing all item parameters (*a*_*i*_s, *b*_*i*_s, ξ_*ic*_s, λ_*ic*_s, $\sigma_{ic}^{2}$s, or ϕ_*ic*_s) with international estimates as derived in step one. Models containing the suffix “_Strong” indicate strong measurement invariance in which item-specific residual variances ($\sigma_{ic}^{2}$s) are allowed to be estimated, instead. Weak measurement invariance models contain the suffix “_Weak.” Here, both the item-specific residual variance ($\sigma_{ic}^{2}$s) and item-time intensity parameters (ξ_*ic*_s) were freely estimated. In this case, however, the mean of the latent speed variable was fixed to 0 for model identification. Lastly, structural measurement invariance (suffix “_Struct”) indicates all time-related parameters are freely estimated (ξ_*ic*_s, λ_*ic*_s, $\sigma_{ic}^{2}$s or ϕ_*ic*_s). For model identification, we fixed the mean and the variance of the latent speed variable to 0 and 1, respectively. We also incorporated a new constraint in model “M3_Struct” to allow the free estimation of the cross-loading parameter (ϕ_*ic*_s). In this case, we constrained the variance of the latent speed to be the same as the estimates from the M1_Full model to make sure that the correlations between *X*_*i*_s and θ will be the same as in model 1 and therefore θ will have similar interpretation as in M1.

**Table 4:** Framework for the estimation of countries' parameters for each analyzed model.

**Model**	**μ_*θc*_**	**$\sigma_{\theta c}^{2}$**	**μ_*τc*_**	**$\sigma_{\tau c}^{2}$**	***a*_*i*_s**	***b*_*i*_s**	**ξ_*ic*_s**	**λ_*ic*_s**	**$\sigma_{ic}^{2}$s**	**ρ_*θτc*_**	**ϕ_*ic*_s**
M1_Full	Free	Free	-	-	*a*_*i*_s M1	*b*_*i*_s M1	-	-	-	-	-
M2_Full	Free	Free	Free	Free	*a*_*i*_s M1	*b*_*i*_s M1	ξ_*i*_s M2	λ_*i*_s M2	$\sigma_{i}^{2}$s M2	Free	-
M2_Strong	Free	Free	Free	Free	*a*_*i*_s M1	*b*_*i*_s M1	ξ_*i*_s M2	λ_*i*_s M2	Free	Free	-
M2_Weak	Free	Free	0	Free	*a*_*i*_s M1	*b*_*i*_s M1	Free	λ_*i*_s M2	Free	Free	-
M2_Struct	Free	Free	0	1	*a*_*i*_s M1	*b*_*i*_s M1	Free	Free	Free	Free	-
M3_Full	Free	Free	Free	Free	*a*_*i*_s M1	*b*_*i*_s M1	ξ_*i*_s M3	λ_*i*_s M3	$\sigma_{i}^{2}$s M3	Free	ϕ_*i*_s M3
M3_Strong	Free	Free	Free	Free	*a*_*i*_s M1	*b*_*i*_s M1	ξ_*i*_s M3	λ_*i*_s M3	Free	Free	ϕ_*i*_s M3
M3_Weak	Free	Free	0	Free	*a*_*i*_s M1	*b*_*i*_s M1	Free	λ_*i*_s M3	Free	Free	ϕ_*i*_s M3
M3_Struct	Free	$\sigma_{\theta c}^{2}$ M1_Full	0	1	*a*_*i*_s M1	*b*_*i*_s M1	Free	Free	Free	0	Free

(1) Since ϕ_ic_s are not freely estimated in the models M3_Full, M3_Strong, and M3_Weak, the correlation between the latent variables is identified. For the M3_Struct model, however, we constrained the variance of the latent speed to be the same as the estimates from the M1_Full model to make sure that the correlations between X_i_s and θ will be the same as in model 1 and therefore θ will have similar interpretation as M1. (2) After obtaining the parameter estimates in the M3_Struct model, the second latent variable (τ^*^) was rotated to match the latent speed variable in M2, and ρ_θτc_ was calculated.

We estimated student abilities using the Expected a Posteriori (EAP) approach (Bock and Mislevy, 1982) and evaluated measurement precision using the EAP-reliability method (Adams, 2005) and the average of the standard errors of the ability estimates. Finally, we computed the Bayesian Information Criterion (BIC) for model selection (Schwarz, 1978).

## 3. Results

We addressed our research questions by assessing the results according to the following three steps: (1) we estimated the overall ability estimates and their level of precision regarding PISA 2012 digital math items by the three measurement models, (2) presented our findings about the invariance of response-time model parameters across items and countries, and (3) showed changes in country-level performance when time-on-task was considered.

### 3.1. RQ1: Overall Performance

We first investigated the model fit for the graded response model. This model was assessed as having a good fit based on its SRMSR (0.036). It also exhibited acceptable fit according to its RMSEA (0.050). We thus concluded that our baseline model had sufficiently good overall fit for continued analyses, including those related to time-on-task variables.

Table 5 shows the overall estimates for student abilities and the measurement precision of these estimates in relation to the PISA 2012 digital math items across the different models. Although there was no substantial difference, M2 and M3 (i.e., the simple-structure hierarchical model and the cross-loadings model, respectively) exhibited increased measurement precision (as captured by larger EAP reliability estimates and smaller average standard errors) when response times were included in the modeling framework.

**Table 5:** Estimated means and variances of students' abilities, EAP reliability and average of the standard errors for the three measurement models.

**Model**	**Mean (${\hat{\mu }}_{\theta }$)**	**Variance (${\hat{\sigma }}_{\theta }^{2}$)**	**EAP reliability**	**Average SE**
M1	0.00	1.00	0.73	0.51
M2	−0.02	1.06	0.77	0.49
M3	−0.02	1.05	0.80	0.45

*International results of the PISA 2012 digital math items*.

### 3.2. RQ2: Measurement Invariance

We investigated measurement invariance of the time-on-task parameters for each country with both M2 and M3. We also calculated the BIC for each individual model and summarized these statistics to identify the level of invariance that best represented the data overall (Table 6). As such, the assumption of invariance of the model's parameters does not hold for most countries and models. Weak measurement invariance were preferred in most of the cases (i.e., there was country-specific heterogeneity in the time intensity (ξ_*i*_) and residual variance ($\sigma_{i}^{2}$) parameters for the time-on-task measurement models).

**Table 6:** Model fit statistics (BIC) by model and country.

**Country**	**M2 models**		**M3 models**	
	**Range BIC**	**Preferred**	**Range BIC**	**Preferred**
ARE	[29647.12–29774.20]	M2_Struct	[29537.07–29815.97]	M3_Weak
AUS	[54700.07–54766.34]	M2_Weak	[54328.41–54415.99]	M3_Full
AUT	[13147.99–13240.82]	M2_Full	[13049.54–13149.53]	M3_Full
BEL	[20621.68–20670.20]	M2_Full	[20487.24–20539.84]	M3_Weak
BRA	[12137.77–12214.90]	M2_Weak	[12091.05–12181.83]	M3_Weak
CAN	[43637.52–43803.38]	M2_Strong	[43440.33–43598.05]	M3_Weak
COL	[21367.18–21653.93]	M2_Weak	[21289.22–21653.15]	M3_Weak
DEU	[13002.93–13065.20]	M2_Full	[12919.52–13010.30]	M3_Full
DNK	[18226.95–18269.57]	M2_Full	[18090.74–18175.80]	M3_Full
ESP	[27485.45–27588.13]	M2_Full	[27287.14–27437.58]	M3_Full
EST	[12629.05–12761.87]	M2_Strong	[12482.02–12625.00]	M3_Strong
FRA	[12890.52–12913.41]	M2_Weak	[12772.27–12824.20]	M3_Weak
HKG	[13527.25–14034.09]	M2_Struct	[13479.78–13752.47]	M3_Struct
HUN	[12181.87–12197.26]	M2_Strong	[12110.29–12155.05]	M3_Strong
IRL	[10701.96–10754.46]	M2_Strong	[10602.46–10685.45]	M3_Strong
ISR	[12298.66–12436.20]	M2_Weak	[12229.09–12370.03]	M3_Weak
ITA	[12674.72–12748.87]	M2_Weak	[12559.46–12616.12]	M3_Full
JPN	[30542.91–31258.25]	M2_Struct	[30320.88–31046.07]	M3_Struct
KOR	[13060.99–13190.37]	M2_Struct	[12977.21–13138.38]	M3_Weak
MAC	[15390.05–15536.21]	M2_Weak	[15250.20–15396.36]	M3_Weak
NOR	[12867.52–12905.24]	M2_Strong	[12784.82–12845.24]	M3_Weak
POL	[12530.05–12563.72]	M2_Full	[12444.90–12511.19]	M3_Full
PRT	[14017.90–14079.03]	M2_Full	[13934.64–14045.98]	M3_Full
QCN	[11640.00–11840.43]	M2_Weak	[11579.94–11665.66]	M3_Struct
RUS	[15790.27–15843.04]	M2_Weak	[15683.03–15754.20]	M3_Weak
SGP	[13129.26–13240.15]	M2_Weak	[13016.65–13090.06]	M3_Strong
SVK	[13976.93–14004.75]	M2_Struct	[13877.89–13903.17]	M3_Full
SVN	[19994.64–20004.67]	M2_Weak	[19942.30–19986.59]	M3_Strong
SWE	[12449.46–12509.05]	M2_Full	[12325.18–12420.05]	M3_Full
TAP	[15235.19–15292.96]	M2_Struct	[15121.61–15158.37]	M3_Full
USA	[11209.98–11277.11]	M2_Weak	[11186.89–11239.37]	M3_Weak
Total BIC	[556031.15–557223.67]	M2_Weak	[552353.33–555491.33]	M3_Weak

*(1) The range indicates the minimum and maximum values of the BIC statistic by country. (2) The suffix “_Full” indicates full measurement invariance (fixing all item parameters to be equal to the international estimates), “_Strong” indicates strong measurement invariance (country-specific residual variances are allowed to be estimated, while time intensity parameters and factor loadings are fixed to be equal to the international estimates), “_Weak” means weak measurement invariance model (country-specific residual variances and country-time intensity parameters are freely estimated, while factor loadings are fixed to be equal to the international estimates), and “_Struct,” structural measurement invariance (wholly fitted time-related parameters, i.e., all time-related parameters are freely estimated in each country)*.

**Figure 2:**
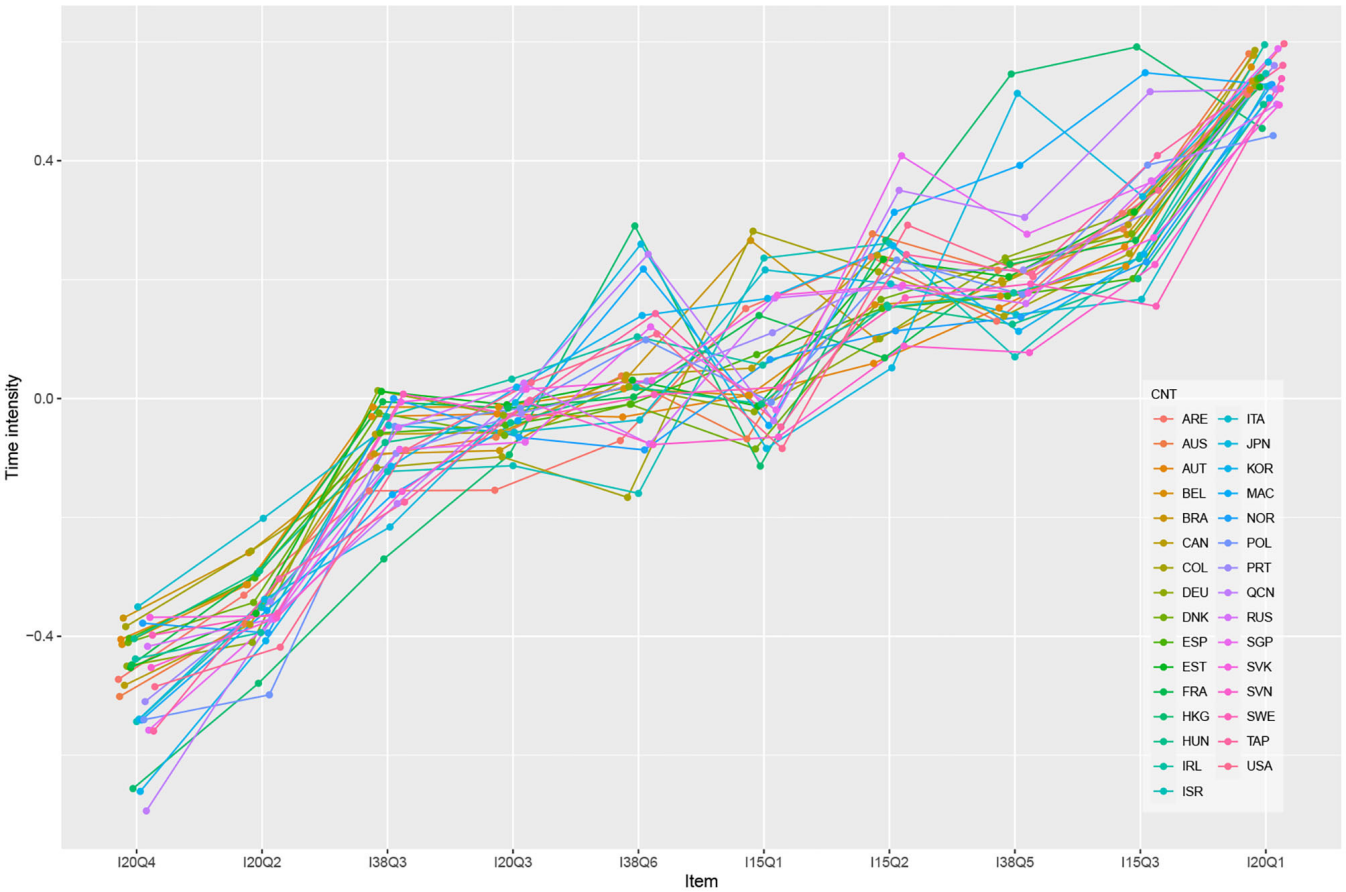
Estimates of the countries' time intensity for model 2—Weak measurement invariance.

To illustrate the differences in the time-on-task measurement model parameters, Figure 2 presents the estimated time-intensity parameters for each item in each country as applied to the preferred model in the simple-structure framework (M2). The graph indicates that students in all analyzed countries placed the most effort into answering the first item, I20Q1, from Unit 20 (Star Point unit). However, the pattern of estimated time-intensity between different items varied according to country. For example, the estimated time intensity of item I38Q06 was larger than that of item I15Q01 for several countries, but the opposite was found for about just as many countries.

### 3.3. RQ3: Country-Level Performance

**Figure 3:**
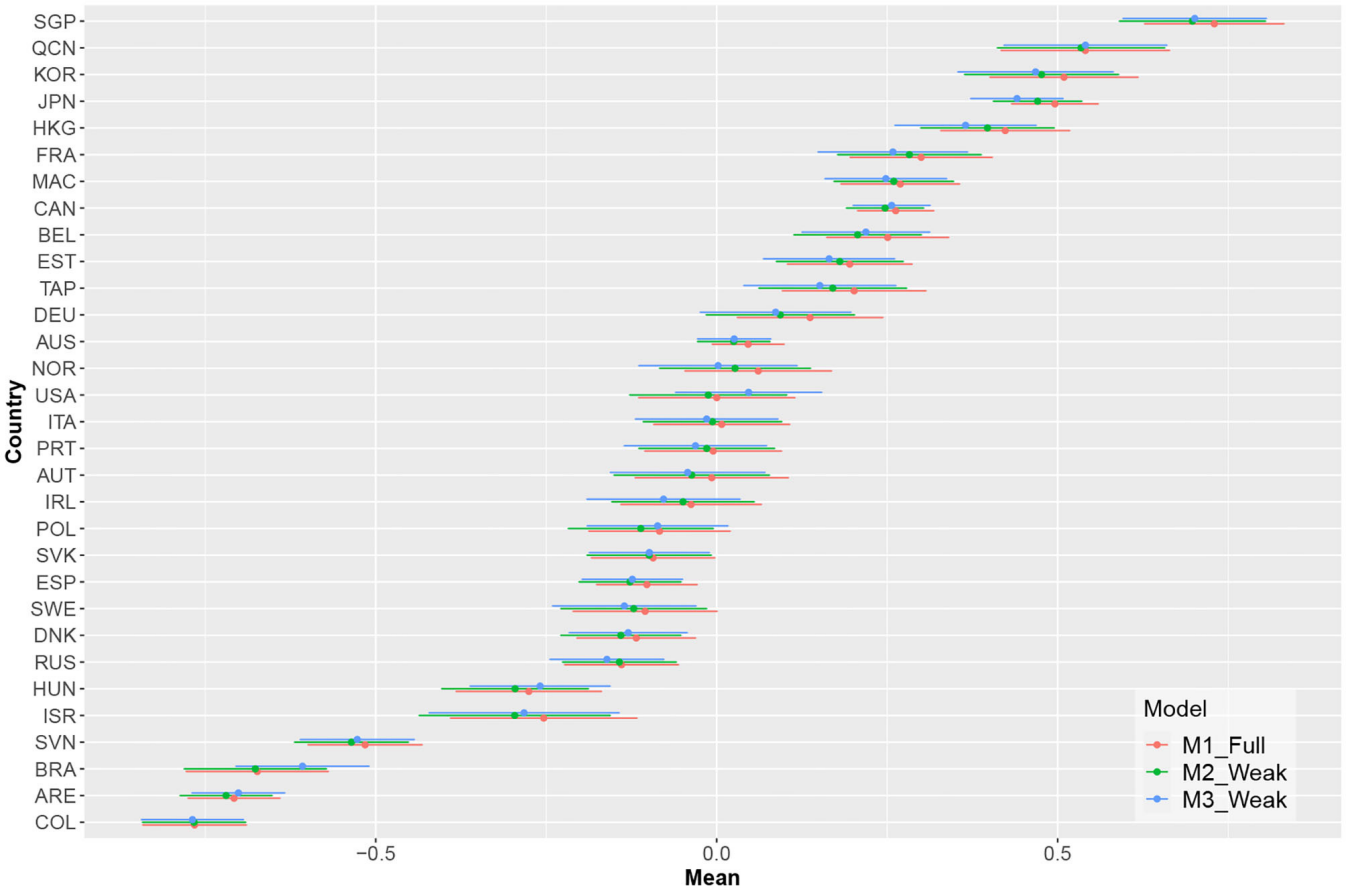
Estimates of the countries' means and their respective confidence intervals for the different models.

Figure 3 shows the estimated country means in computer-based mathematical literacy and the associated confidence intervals for the three measurement models. The estimated means did not show substantial discrepancies for the analyzed countries between the different models.

**Figure 4:**
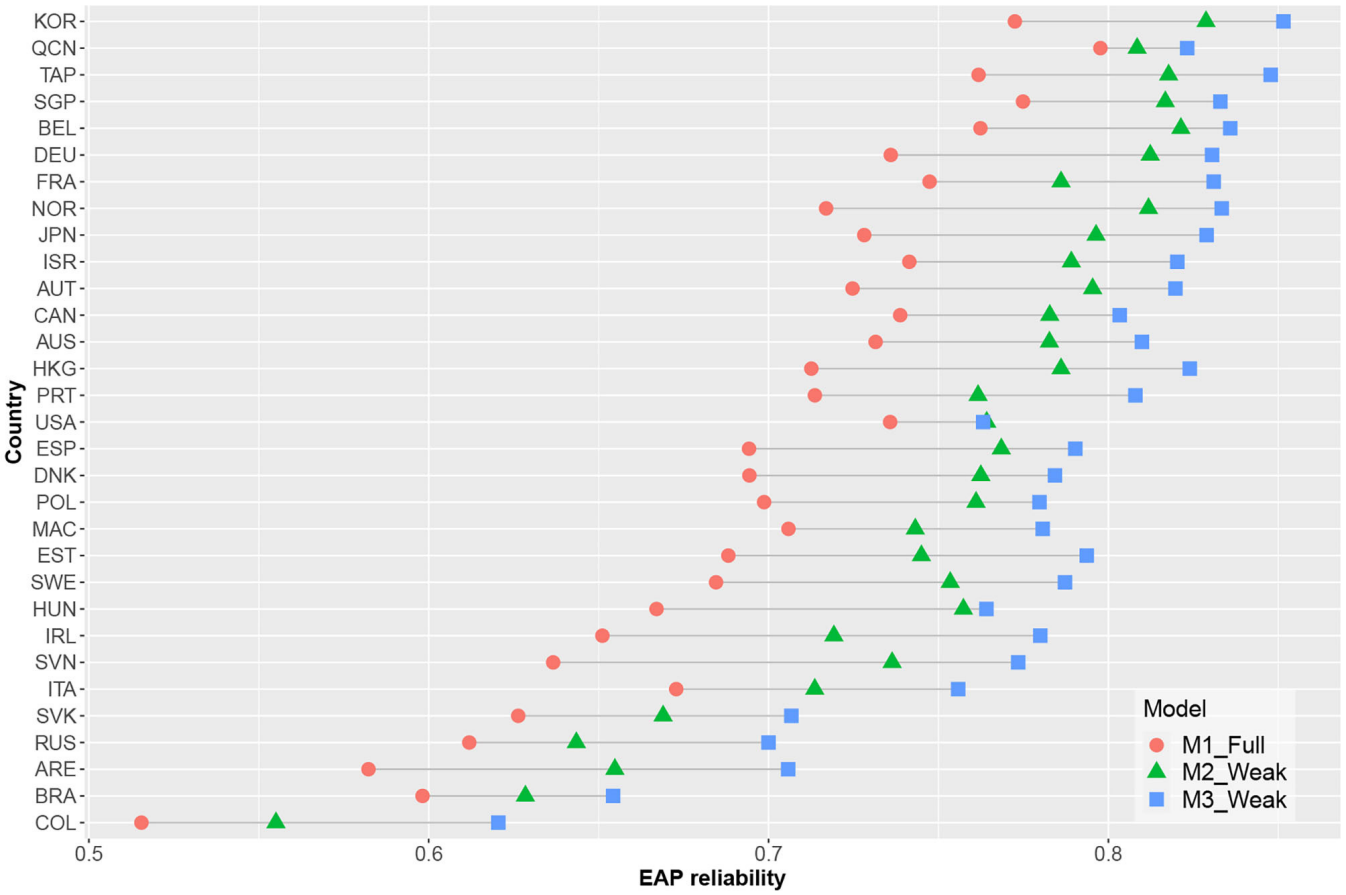
EAP reliabilities estimates per country and model.

**Figure 5:**
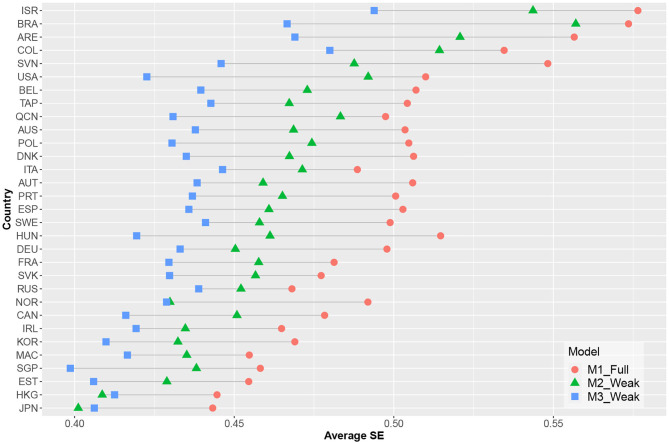
Average standard errors of abilities' estimates per country and model.

Figure 4 shows the estimated reliability of the EAP ability estimates for each country. Measurement precision increased for all countries when time-on-task variables were included; here, the model containing cross-loadings had the highest estimated EAP reliability. As illustrated in Figure 5, there was a decrease in average standard errors for ability estimates when time-on-task variables were included.

**Figure 6:**
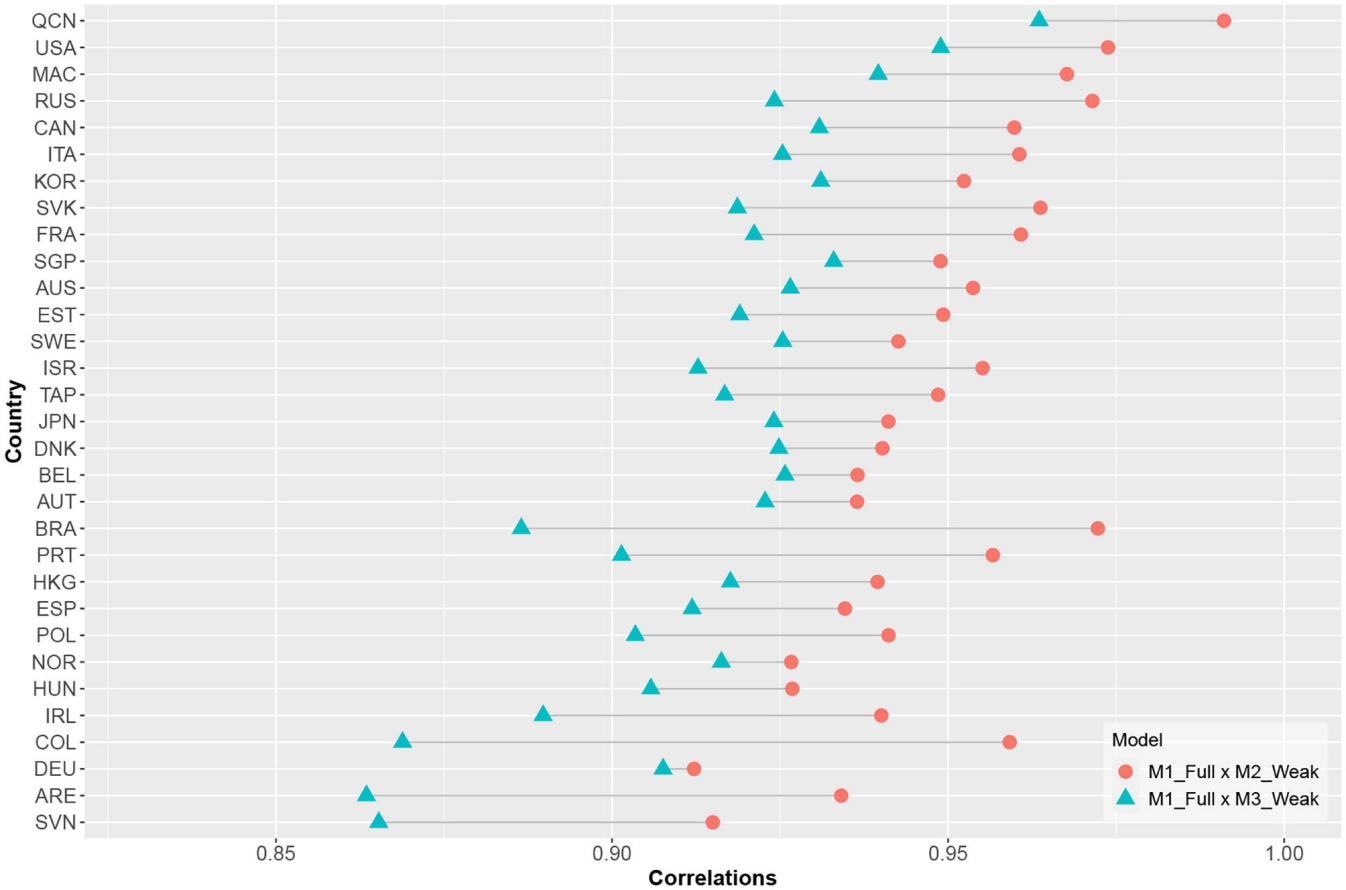
Correlations between EAP estimates.

Figure 6 shows the correlations between EAP ability estimates from the baseline model and from those including time-on-task variables. Ability estimates from models that included cross-loadings generally had lower correlations with the baseline model-based ability estimates as compared to models that did not include cross-loadings. This indicates that the ability estimates from model 3 captured an additional source of information. However, this may not have reflected the test objective (i.e., estimating student computer-based mathematical literacy).

## 4. Summary and Discussion

This study examined the extent to which inferences about ability in large-scale educational assessments were affected by and improved by including time-on-task information in the statistical analyses. This issue was specifically explored using data from the PISA 2012 Computed-Based Assessment of Mathematics. In line with statistical theory, model-based measurement precision (as captured by the EAP reliability estimates) improved when using the standard hierarchical model as opposed to the response accuracy only model for each of the 31 considered countries that participated in the PISA program. This increase was notable for most countries, with many showing increases in estimated EAP reliability at or above 0.05. If such a version of the hierarchical model can adequately capture the data structure, then this suggests it can also provide a notable increase in precision over the default response-accuracy only models.

For practically all countries, model-based measurement precision was further increased when using the extended version of the hierarchical model, which allowed a direct link between response times and ability by including cross-loadings (i.e., rather than using the standard hierarchical model). This model successfully extended the hierarchical model by considering overall response speed as relevant to the estimated ability while also allowing individual item-response times to be linked to said ability if such patterns were present in the data. Thus, the model allowed time-on-task to provide more collateral information when estimating ability than was possible when using the standard hierarchical model. This increased precision was also notable for most countries (generally between 0.02 and 0.03). However, the increase was generally less sizable than those obtained by using the hierarchical model instead of a response-accuracy only model. Thus, the biggest gain in precision was already obtained by using a simple-structure hierarchical model; extending the model by incorporating cross-loadings generally only resulted in modest additional gains.

We investigated the extent to which time-on-task parameters could be considered invariant across countries for both the simple-structure hierarchical model and the extension that included cross-loadings. The results suggested that only weak measurement invariance existed. As such, full or strong measurement invariance did not hold. That is, our findings suggest that countries may differ both in item time-intensity (capturing how much time respondents generally spent on items) and the item-specific variability of the response times (i.e., the degree to which respondents differed in the amounts of time they spent on particular items). This suggests relevant differences between countries in regard to how respondents approached items as well as in their response processes.

Measurement precision improved for all countries when using the selected versions of M2 and M3 (i.e., over the precision levels obtained using M1). Since changing the model used to analyse the data may also affect model-based inferences, we also analyzed the extent to which such inferences would be affected by these changes. Here, no country showed a substantial change in estimated mean, thus suggesting that the overall assessment of proficiency levels for different countries was not heavily affected by a model change. However, the estimated correlations between the individual ability estimates obtained using M1, M2, and M3 showed small deviations from 1 for many countries, suggesting that the ability being estimated does not overlap perfectly across the three models. The differences between M1 and M3 were most notable in this regard. That is, they generally resulted in the lowest correlations between ability estimates. It is thus not surprising that these two models had the lowest correlation; they also had the largest differences in modeling structure. However, one should carefully consider which of the models best operationalizes the specific ability that will be estimated. Additional validation research is thus needed to determine whether the inclusion of time-on-task information results in overall improved measurement quality.

## Data Availability Statement

Publicly available datasets were analyzed in this study. This data can be found here: https://www.oecd.org/pisa/pisaproducts/database-cbapisa2012.htm.

## Author Contributions

DRC: conceptualization. DRC, MB, JT, and BA: methodology and paper writing. DRC: data analysis. All authors contributed to the article and approved the submitted version.

## Conflict of Interest

The authors declare that the research was conducted in the absence of any commercial or financial relationships that could be construed as a potential conflict of interest.

## Footnotes

^1^We use “country” as a generic term referring to all countries and economies participating in the PISA study. A list of the countries codes is displayed in the Supplementary Material.

^2^The logistic function was used here because it is more common than the cumulative normal function used in IRT applications, including for large-scale international assessments such as PISA.

## Supplementary Material

TABLE S1 List of country codes.

## References

[B1] Adams R. J. (2005). Reliability as a measurement design effect. Stud. Educ. Eval. 31, 162–172. 10.1016/j.stueduc.2005.05.008

[B2] Birnbaum A. L. (1968). “Some latent trait models and their use in inferring an examinee's ability,” in Statistical Theories of Mental Test Scores, eds Lord F. M. Novick M. R. (Reading: Addison-Wesley), 397–479.

[B3] Bock R. D. Mislevy R. J. (1982). Adaptive EAP estimation of ability in a microcomputer environment. Appl. Psychol. Meas. 6, 431–444. 10.1177/014662168200600405

[B4] Bolsinova M. Tijmstra J. (2018). Improving precision of ability estimation: getting more from response times. Brit. J. Math. Stat. Psychol. 71, 13–38. 10.1111/bmsp.12104 28635139

[B5] Chalmers R. P. (2012). mirt: a multidimensional item response theory package for the R environment. J. Stat. Softw. 48, 1–29. 10.18637/jss.v048.i06

[B6] Goldhammer F. Klein Entink R. H. (2011). Speed of reasoning and its relation to reasoning ability. Intelligence 39, 108–119. 10.1016/j.intell.2011.02.00125824536

[B7] Goldhammer F. Naumann J. Greiff S. (2015). More is not always better: the relation between item response and item response time in Raven's matrices. J. Intell. 3, 21–40. 10.3390/jintelligence3010021

[B8] Goldhammer F. Naumann J. Stelter A. Tóth K. Rölke H. Klieme E. (2014). The time on task effect in reading and problem solving is moderated by task difficulty and skill: insights from a computer-based large-scale assessment. J. Educ. Psychol. 106, 608–626. 10.1037/a0034716

[B9] Goldhammer F. Zehner F. (2017). What to make of and how to interpret process data. Measurement 15, 128–132. 10.1080/15366367.2017.1411651

[B10] Greiff S. Wüstenberg S. Avvisati F. (2015). Computer-generated log-file analyses as a window into students' minds? A showcase study based on the PISA 2012 assessment of problem solving. Comput. Educ. 91, 92–105. 10.1016/j.compedu.2015.10.018

[B11] Hu L. T. Bentler P. M. (1999). Cutoff criteria for fit indexes in covariance structure analysis: Conventional criteria versus new alternatives. Struct. Equat. Model. 6, 1–55. 10.1080/10705519909540118

[B12] Klein Entink R. H. Fox J.-P. van der Linden W. J. (2009). A multivariate multilevel approach to the modeling of accuracy and speed of test takers. Psychometrika 74:21. 10.1007/s11336-008-9075-y 20037635 PMC2792348

[B13] Kyllonen P. Zu J. (2016). Use of response time for measuring cognitive ability. J. Intell. 4:14. 10.3390/jintelligence4040014

[B14] Molenaar D. Tuerlinckx F. van der Maas H. L. (2015). A generalized linear factor model approach to the hierarchical framework for responses and response times. Brit. J. Math. Stat. Psychol. 68, 197–219. 10.1111/bmsp.12042 25109494

[B15] Muthén B. Muthén L. (2012). Mplus Computer Program Version 7.3. Los Angeles, CA: Mplus.

[B16] Naumann J. Goldhammer F. (2017). Time-on-task effects in digital reading are non-linear and moderated by persons' skills and tasks' demands. Learn. Individ. Diff. 53, 1–16. 10.1016/j.lindif.2016.10.002

[B17] OECD (2010). PISA Computer-Based Assessment of Student Skills in Science. Technical report, Paris. 10.1787/9789264082038-en

[B18] OECD (2011). PISA 2009 Results: Students On Line: Digital Technologies and Performance. OECD Publishing, Paris. 10.1787/9789264112995-en

[B19] OECD (2014a). Pisa 2012 Results: Creative Problem Solving: Students' Skills in Tackling Real-Life Problems. Vol. 5. Technical report, Paris.

[B20] OECD (2014b). PISA 2012 Technical Report. OECD Publishing, Paris.

[B21] OECD (2014c). Results: What Students Know and Can Do Student Performance in Mathematics, Reading and Science. OECD Publishing, Paris.

[B22] OECD (2019). Beyond Proficiency: Using Lof Files to Understand Respondent Behaviour in the Survey of Adult Skills. OECD Publishing, Paris. 10.1787/0b1414ed-en

[B23] Ranger J. (2013). A note on the hierarchical model for responses and response times in tests of van der linden (2007). Psychometrika 78, 538–544. 10.1007/s11336-013-9324-6 25106399

[B24] Reis Costa D. Leoncio W. (2019). LOGAN: Log File Analysis in International Large-Scale Assessments. (R package version 1.0.0). Available online at: https://cran.r-project.org/web/packages/LOGAN/index.html

[B25] Rijmen F. Tuerlinckx F. De Boeck P. Kuppens P. (2003). A nonlinear mixed model framework for item response theory. Psychol. Methods 8, 185–205. 10.1037/1082-989X.8.2.185 12924814

[B26] Samejima F. (1969). Estimation of latent ability using a response pattern of graded scores. Psychometrika. 34, 1–97. 10.1007/BF03372160

[B27] Schwarz G. (1978). Estimating the dimension of a model. Ann. Stat. 6, 461–464. 10.1214/aos/1176344136

[B28] Skrondal A. Rabe-Hesketh S. (2004). Generalized Latent Variable Modeling:Multilevel, Longitudinal and Structural Equation Models. Boca Raton, FL: Chapman & Hall/CRC.

[B29] van der Linden W. J. (2007). A hierarchical framework for modeling speed and accuracy on test items. Psychometrika 72:287. 10.1007/s11336-006-1478-z

[B30] von Davier M. Sinharay S. (2013). “Analytics in international large-scale assessments: item response theory and population models,” in Handbook of International Large-Scale Assessment: Background, Technical Issues, and Methods of Data Analysis, eds Rutkowski L. von Davier M. Rutkowski D. (Boca Raton, FL: CRC Press), 155–174.

