# Supplementary material for: Improving the Precision of Ability Estimates Using Time-On-Task Variables: Insights From the PISA 2012 Computer-Based Assessment of Mathematics
Source: Front Psychol. 2021 Mar 19;12:579128. doi: 10.3389/fpsyg.2021.579128 (PMC8017127; doi:10.3389/fpsyg.2021.579128)
Supplement: TABLE S1 — List of country codes. [file Data_Sheet_1.PDF]

Supplementary Material

1 SUPPLEMENTARY TABLE

Table S1. List of country codes

ISO code	Country/Economy
ARE	United Arab Emirates
AUS	Australia
AUT	Austria
BEL	Belgium
BRA	Brazil
CAN	Canada
COL	Colombia
DEU	Germany
DNK	Denmark
ESP	Spain
EST	Estonia
FRA	France
HKG	Hong Kong-China
HUN	Hungary
IRL	Ireland
ISR	Israel
ITA	Italy
JPN	Japan
KOR	Korea
MAC	Macao-China
NOR	Norway
POL	Poland
PRT	Portugal
QCN	Shanghai-China
RUS	Russian Federation
SGP	Singapore
SVK	Slovak Republic
SVN	Slovenia
SWE	Sweden
TAP	Chinese Taipei
USA	United States
